# Association of visual impairment with cognitive decline among older adults in Taiwan

**DOI:** 10.1038/s41598-021-97095-9

**Published:** 2021-09-02

**Authors:** I.-Mo Fang, Yi-Jen Fang, Hsiao-Yun Hu, Shih-Han Weng

**Affiliations:** 1 Department of Ophthalmology, Taipei City Hospital, Zhongxiao Branch, No. 87, Tonde Road, Nankang District, Taipei, Taiwan, ROC; 2 Department of Ophthalmology, National Taiwan University Hospital, Taipei, Taiwan, ROC; 3 Department of Special Education, University of Taipei, Taipei, Taiwan, ROC; 4 Center for Environmental Medicine, Kaohsiung Medical University, Kaohsiung, Taiwan, ROC; 5 National Institute of Environmental Health Sciences, National Health Research Institutes, Zhunan, Taiwan, ROC; 6 Digestive Disease Center, Show-Chwan Memorial Hospital, Changhua, Taiwan, ROC; 7 Department of Education and Research, Taipei City Hospital, Taipei, Taiwan, ROC; 8 Institute of Public Health, National Yang Ming Chiao Tung University, Taipei, Taiwan, ROC; 9 University of Taipei, Taipei, Taiwan, ROC

**Keywords:** Health care, Risk factors

## Abstract

This study investigated the association between visual impairment and cognitive decline among the elderly in Taiwan. The data were obtained from a government-sponsored, annual physical examination program for elderly citizens ≥ 65 years in Taipei City during 2005–2012. Distance presenting visual acuity was measured using the Snellen chart. Visual impairment was classified into low vision and blindness. The Short Portable Mental Status Questionnaire (SPMSQ) was selected to measure cognitive decline. The confounding factors including age, sex, sociodemographic factors: living status, marital status, education level, health behaviors: smoking, alcohol consumption, betel nut chewing, and physical comorbidities: BMI, hypertension, diabetes, cholesterol and triglyceride were collected for analysis. We recruited 105,208 participants and 4542 (4.3%) have abnormal SPMSQ. The abnormal SPMSQ had significantly higher prevalence of low vision (44.52% vs 18.79%) and blindness (8.89% vs 0.93%) compared with normal SPMSQ. The hazard ratios of abnormal SPMSQ in low vision and blindness were 2.34 (95% CI 2.17–2.52), and 5.13 (95% CI 4.50–5.85), after adjustment for confounders. In conclusion, visual impairment was independently and significantly associated with greater incident cognitive decline among elderly Asian people. Prevention of visual impairment may help to reduce the incidence of cognitive decline in the aged Asian population.

## Introduction

Cognitive decline, including mild cognitive impairment (MCI) and dementia, is characterized by a decline from a previously attained cognitive level^1^. Cognitive decline will reduce the quality of life and increase the mortality rate of the elderly, and raise the medical and social burdens^2,3^. A nationwide survey of Taiwanese population aged 65 or above in 2014 showed the prevalence of all-cause dementia was 8.04% and age-adjusted prevalence of MCI was 18.76%^4^. As life expectancy increases, the number of people with cognitive decline can be expected to increase rapidly. Therefore, identifying the possible modifiable risk factors of cognitive decline and preventing or even treating them is a very important issue in an aging society.

At present, many risk factors for cognitive decline in the elderly include: medical disorders, education, lifestyle and nutrition have been identified^5–7^. It is generally believed that social and daily activities, such as face-to-face contact, reading and exercise, can enhance cognitive reserve and therefore are believed to reduce the chance of cognitive decline^8,9^. In theory, visual impairment will hinder these cognitive stimulation activities, which can easily lead to cognitive decline. Although some previous studies have revealed the relationship between visual impairment and cognitive decline, most of these studies have been conducted in western populations^10–12^. There is still a lack of large-scale Asian and longitudinal studies related to vision and cognitive decline.

The Short Portable Mental State Questionnaire (SPMSQ) is a well-established cognitive screening tool for diagnosing cognitive decline^13^. SPMSQ only requires oral inquiry, so medical staff and researchers can easily use it with little training^14^. Therefore, it is a good tool for large-scale screening of cognitive decline.

This study had two purposes: the main purpose was to explore the association between visual impairment and cognitive decline in a population-based cohort of Asian elderly population. The second purpose was to determine the risk factors for cognitive decline in social status, education level, physical factors, habits and nutritional status among Asian elderly.

## Methods

### Study population

In this retrospective cohort study, the data were collected from a standard, annul physical examination program for the elderly population, conducted by the Taipei City government during 2005–2012. Taipei citizens older than 65 years old are eligible to participate voluntarily and are encouraged to visit annually at no cost. As for aboriginal citizens, the eligibility criteria are extended to 55 years of age or older. The demographic and lifestyle data (e.g., marital status, education level, smoking status, betel nut chewing status and alcohol consumption) were collected through self-administered questionnaires^15^. Chewing betel nut has long been a habit of Taiwanese natives and blue-collar workers, and is associated with many diseases such as cancer, ulcers and metabolic syndrome^16^. For participants who have participated in the program more than once, only the data from the first visit were used for analysis.

During the medical checkup, blood pressure measurement, blood sample collection, and laboratory analyses were performed for each participant. The initial database included 305,549 original data during 2005–2012. We excluded 892 data aged less than 65 years and further excluded missing data regarding presenting visual acuity (n = 9492), age (n = 95), living status (n = 6), BMI (n = 575), marital status (n = 1533), education level (n = 13,515), smoking status (n = 234), alcohol consumption (n = 256), betel nut chewing (n = 419), hypertension (n = 174), blood sugar level (n = 256), blood cholesterol level (n = 339), blood triglycerol level (n = 328), blood albumin level (n = 6131) and blood globulin level (n = 5959). The final analytical sample comprised 105,208 participants. The data regarding participant identification were removed to ensure participant anonymity throughout the study period. The study adhered to the tenets of the Declaration of Helsinki and was approved by the Institutional Review Board of Taipei City Hospital (IRB No.: TCHIRB-10703110-W), and written informed consent was obtained from all patients.

### Visual acuity measurement and definition of visual impairment

Distance presenting visual acuity for each eye was measured under normal luminance using the Snellen chart at a distance of 6 m (20 feet). Participants were asked to wear their usual distance vision correction, if any. Visual acuity of the better-seeing eye was used to characterize visual impairment status. For participants with visual acuity data in only one eye, visual acuity of the lone measured eye was regarded as the visual acuity of the better-seeing eye. According to the new World Health Organization (WHO) classification for blindness and visual impairment, visual impairment was classified into: (1) mild visual impairment, which were defined as a presenting visual acuity in the better-seeing eye worse than 6/12 to 6/18; (2) moderate visual impairment: worse than 6/18 to 6/60; (3) severe impairment: worse than 6/60 to 3/60; and (4) blindness: worse than 3/60. In our study, moderate and severe visual impairment were defined as low vision^17^.

### Other confounding variables

Baseline data were collected, which included age (65–74 years; 75–84 years and ≧ 85 years), sex, living status (living alone and not living alone), marital status (single/separated; married/cohabiting; divorce/widowed), education level (illiterate; elementary/junior high school; and above senior high school), smoking status in the past 6 months (the participants who had reported smoking every day or some days in the past 6 months were defined as smokers; those who had never smoked in the past 6 months were defined as nonsmokers), alcohol consumption in the past 6 months (the participants who had reported drinking every day or some days in the past 6 months were defined as drinkers; those who did not drink alcohol in the past 6 months were defined as nondrinkers) and betel nut chewing (the participants who had reported chewing betel nut every day or some days in the past 6 months were defined as betel nut chewer; those who did not chew betel nut in the past 6 months were defined as nonchewer). Both height and weight were measured during the examination by using standardized procedures. BMI-based categories were defined as underweight (BMI < 18.5), normal weight (BMI 18.5–24), overweight (BMI > 24.0–27), and obese (BMI > 27). Diabetes mellitus was defined as either fasting blood sugar ≧ 126 mg/dL, self-report of physician- diagnosed diabetes mellitus, or the use of hypoglycemic medications. Hypertension was defined as either blood pressure > 140/90 mm Hg, self-report of physician-diagnosed hypertension, or the use of antihypertension medications. Hyperlipidemia was defined as either triglyceride ≧ 200 mg/dL, and total cholesterol ≧ 200 mg/dL, self-report of physician-diagnosed hyperlipidemia, or the use of lipid-lowering medications. The definition of these variables was based on the article published by Hu et al.^15^.

### Measures of dependent variables

In this study, cognitive function was assessed by using the validated Short Portable Mental Status Questionnaire (SPMSQ). It is a widely used scale for assessing the mental status of older adults. The scale contains ten item questions: items test orientation to time and place, memory, current event information (date, day of the week, name of this place, telephone number, date of birth, age, name of current president and previous president, mother’s maiden name), and calculation (subtract 3 s starting with number 20). The SPMSQ error score comes from the amount of errors based on the above 10-item list by coding errors as “1” and correct answers as “0”. The total score is computed and it ranges from 0 to 10^18^. A score with 0–2 errors indicates no cognitive decline. Abnormal SPMSQ is defined as an error score equal to or greater than 3, indicating potential cognitive decline.

### Statistical analyses

Baseline characteristics of participants were compared according to SPMSQ score (normal SPMSQ and abnormal SPMSQ) using a t-test for continuous variables and the chi-square test for categorical variables. Multivariate Cox proportional hazard regression models were applied to determine the association between distance presenting visual acuity (normal, low vision and blindness) and cognitive decline, as demonstrated by abnormal SPMSQ after controlling for all other confounding factors, including age, sex, sociodemographic factors: living status, marital status, education level, health behaviors: smoking, alcohol consumption, betel nut chewing, and physical comorbidities: BMI, hypertension, diabetes, cholesterol and triglyceride. The effects of individual variables were examined in univariate models. Results were presented as hazard ratios (HRs) with 95% confidence interval. Cumulative incidence of abnormal SPMSQ was analyzed using the Kaplan–Meier method, and the differences between the curves of participants with normal vision, low vision and blindness were calculated using the 2-tailed logrank test. The time of entry was the initial examination date (between 2005 and 2012), and the time of exit was the end of follow-up (December 31, 2012) or the date of having abnormal SPMSQ, if earlier. Subgroup analyses were performed to calculate the HRs for abnormal SPMSQ among participants with low vision and blindness compared with participants with normal vision. All p-value were two-sided, and values < 0.05 were considered statistically significant. We conducted all analyses by using SAS (version 9.3; SAS Institute, Inc., Cary, NC) statistical software packages.

## Results

### Participant characteristics

**Figure 1:**
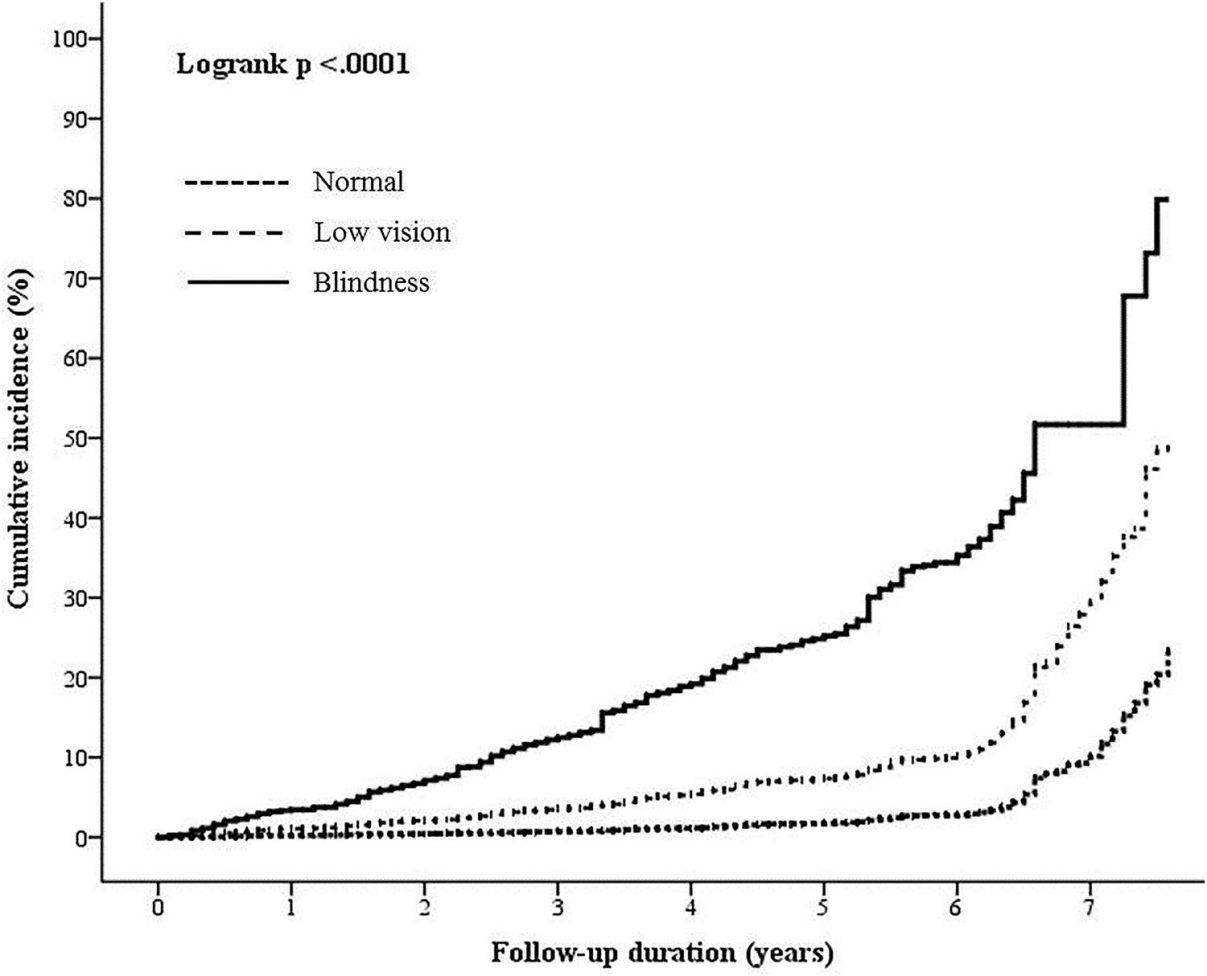
Seven-year cumulative incidences of potential cognitive decline, as demonstrated by abnormal SPMSQ scores among normal, low vision and blindness participants. The blindness participants had the highest incidences of cognitive declines and all of log-rank test were statistically significant (p for trend < 0.0001).

The mean age of the participants was 75.23 ± 6.91 years. During the 7-year study period, 305,549 person-years of follow-up were recorded, with an average follow-up period of 53.72 ± 23.21 months. During the follow-up period, 4542 (4.32%) of the elderly participants had abnormal SPMSQ score. Table 1 summarizes the characteristics of normal and abnormal SPMSQ participants. Among participants with abnormal SPMSQ score, 8.89% were blindness and 44.52% were low vision, whereas 0.93% was blindness and 18.79% were low vision in normal SPMSQ group. A statistically significant difference in visual acuity distribution was found between normal and abnormal SPMSQ group (p < 0.0001, chi-square test). Figure 1 showed the seven-year cumulative incidence curve for cognitive declines, as demonstrated by abnormal SPMSQ, among normal, low vision and blindness participants. The cumulative incidences for abnormal SPMSQ among normal, low vision and blindness participants were 10.15%, 29.53% and 51.67%, respectively. The blindness participants had the highest incidence of cognitive declines and all of logrank test were statistically significant (p for trend < 0.0001, logrank test).

**Table 1:** Characteristics of normal and abnormal SPMSQ participants.

Factors	SPMSQ		p-value
	Normal (N = 100,655)	Abnormal (N = 4542)	
**Sight**			< 0.0001
Normal sight	80,803 (80.28)	2116 (46.59)	
Low vision	18,918 (18.79)	2022 (44.52)	
Blindness	934 (0.93)	404 (8.89)	
**Age**			< 0.0001
65–74	51,070 (50.78)	930 (20.53)	
75–84	40,024 (39.80)	2095 (46.24)	
Elder than 85	9477 (9.42)	1506 (33.24)	
**Gender**			< 0.0001
Male	51,489 (51.15)	1845 (40.62)	
Female	49,166 (48.85)	2697 (59.38)	
**Living status**			< 0.0001
Not living alone	95,086 (94.47)	4352 (95.82)	
Living alone	5563 (5.53)	190 (4.18)	
**Marriage status**			< 0.0001
Married/cohabitation	74,173 (74.74)	2402 (54.31)	
Divorce/widowed	16,450 (16.58)	1263 (28.56)	
Unmarried/separated	8619 (8.68)	758 (17.14)	
**Education level**			< 0.0001
Illiterate	24,909 (28.29)	489 (13.49)	
Elementary/junior high	56,211 (63.83)	1971 (54.39)	
Above senior high school	6941 (7.88)	1164 (32.12)	
**Smoking**			0.0004
No	94,038 (93.62)	4297 (94.92)	
Yes	6409 (6.38)	230 (5.08)	
**Drinking**			< 0.0001
No	98,235 (97.82)	4477 (98.94)	
Yes	2192 (2.18)	48 (1.06)	
**Betel nut**			0.310
No	99,837 (99.57)	4503 (99.67)	
Yes	434 (0.43)	15 (0.33)	
**BMI**			< 0.0001
Under 18.5	3998 (3.99)	425 (9.85)	
18.5–24	45,219 (45.08)	2022 (46.85)	
24–27	31,521 (31.42)	1104 (25.58)	
Over 27	19,568 (19.51)	765 (17.72)	
**Hypertension**			0.201
Normal	62,319 (62.01)	2762 (61.07)	
Abnormal	38,181 (37.99)	1761 (38.93)	
**Blood sugar level**			0.797
Normal	62,163 (61.89)	2781 (61.70)	
Abnormal	38,271 (38.11)	1726 (38.30)	
**Cholesterol**			0.013
Normal	58,884 (58.67)	2723 (60.54)	
Abnormal	41,477 (41.33)	1775 (39.46)	
**Triglyceride**			0.0002
Normal	81,281 (80.98)	3542 (78.76)	
Abnormal	19,092 (19.02)	955 (21.24)	
**Alb**			< 0.0001
Normal	90,584 (95.68)	3810 (86.71)	
Abnormal	4089 (4.32)	584 (13.29)	
**Gl**			0.678
Normal	72,351 (76.29)	3367 (76.56)	
Abnormal	22,492 (23.71)	1031 (23.44)

The hazard ratios (HRs) for cognitive decline as demonstrated by abnormal SPMSQ from Cox regression models were shown in Table 2. Univariate Cox regression analyses revealed that visual impairment, an older age, female sex, not living alone, single marriage status, low education, no drinking, underweight, abnormal TG level and abnormal albumin level increased the risk of cognitive decline (p < 0.0001).

**Table 2:** Univariate Cox regression analysis of factors associated with potential cognitive decline as demonstrated by abnormal SPMSQ.

Factors	Univariate	
	Hazard ratio (95% CI)	p-value
**Sight**		
Normal	1	
Low vision	3.64 (3.43–3.87)	< 0.0001
Blindness	12.8 (11.51–14.24)	< 0.0001
**Age**		
65–74	1	
75–84	1.74 (1.61–1.88)	< 0.0001
Elder than 85	4.79 (4.41–5.2)	< 0.0001
**Gender**		
Female	1	
Male	0.61 (0.58–0.65)	< 0.0001
**Living status**		
Not living alone	1	
Living alone	0.66 (0.57–0.76)	< 0.0001
**Marriage status**		
Married/cohabitation	1	
Divorce/widowed	2.29 (2.14–2.45)	< 0.0001
Unmarried/separated	3.04 (2.8–3.3)	< 0.0001
**Education level**		
Above senior high school	1	
Elementary/junior high	1.83 (1.65–2.02)	< 0.0001
Illiterate	7.92 (7.12–8.8)	< 0.0001
**Smoking**		
No	1	
Yes	0.91 (0.8–1.04)	0.177
**Drinking**		
No	1	
Yes	0.48 (0.36–0.64)	< 0.0001
**Betel nut**		
No	1	
Yes	1.02 (0.62–1.7)	0.93
**BMI**		
18.5–24	1	
Under 18.5	2.25 (2.03–2.5)	< 0.0001
24–27	0.79 (0.73–0.85)	< 0.0001
Over 27	0.89 (0.82–0.97)	0.007
**Hypertension**		
Normal	1	
Abnormal	0.99 (0.93–1.05)	0.675
**Diabetes**		
Normal	1	
Abnormal	1 (0.94–1.06)	0.948
**Cholesterol**		
Normal	1	
Abnormal	0.98 (0.92–1.04)	0.528
**Triglyceride**		
Normal	1	
Abnormal	1.17 (1.09–1.26)	< 0.0001
**Alb**		
Normal	1	
Abnormal	3.18 (2.92–3.47)	< 0.0001
**Gl**		
Normal	1	
Abnormal	1 (0.93–1.07)	1

After control for other covariates, participants with visual impairment showed significantly higher HRs for cognitive decline (low vision: HR: 2.34, 95% confidence interval (CI) 2.17–2.52; blindness: HR: 5.13, 95% CI 4.5–5.85) (Table 3). In addition, multivariate Cox proportional hazard analysis identified an older age (75–84 years: HR = 1.54, 95% CI 1.4–1.69; elder than 85 years: HR = 3.1, 95% CI 2.79–3.45), female sex (HR = 1.37, 95% CI 1.27–1.47), single marriage status (divorce/widowed: HR = 1.49, 95% CI 1.37–1.62; unmarried/separated : HR = 2.49, 95% CI 2.25–2.75), low education (elementary/junior high: HR = 1.62, 95% CI 1.45–1.81; illiterate: HR = 4.64, 95% CI 4.1–5.24), underweight (BMI under 18.5: HR = 1.60, 95% CI 1.42–1.81), hypertension (HR = 1.12, 95% CI 1.04–1.20), abnormal blood TG level (HR = 1.21, 95% CI 1.11–1.32) and abnormal blood albumin level (HR = 2.59, 95% CI 2.33–2.89) as an independent risk factors for cognitive decline. Living alone and drinking were protective factors for cognitive decline (HR = 0.43, 95% CI 0.37–0.51 for living alone; HR = 0.61, 95% CI 0.44–0.86 for drinking habits).

**Table 3:** Multivariate Cox proportional model of factors associated with potential cognitive decline as demonstrated by abnormal SPMSQ.

Factors	Multivariate	
	Hazard ratio (95% CI)	p-value
**Sight**		
Normal	1	
Low vision	2.34 (2.17–2.52)	< 0.0001
Blindness	5.13 (4.5–5.85)	< 0.0001
**Age**		
65–74	1	
75–84	1.54 (1.4–1.69)	< 0.0001
Elder than 85	3.1 (2.79–3.45)	< 0.0001
**Gender**		
Male	1	
Female	1.37 (1.27–1.47)	< 0.0001
**Living status**		
Not living alone	1	
Living alone	0.43 (0.37–0.51)	< 0.0001
**Marriage status**		
Married/cohabitation	1	
Divorce/widowed	1.49 (1.37–1.62)	< 0.0001
Unmarried/separated	2.49 (2.25–2.75)	< 0.0001
**Education level**		
Above senior high school	1	
Elementary/junior high	1.62 (1.45–1.81)	< 0.0001
Illiterate	4.64 (4.1–5.24)	< 0.0001
**Smoking**		
No	1	
Yes	1.02 (0.87–1.2)	0.819
**Drinking**		
No	1	
Yes	0.61 (0.44–0.86)	0.004
**Betel nut**		
No	1	
Yes	1.51 (0.86–2.68)	0.154
**BMI**		
18.5–24	1	
Under 18.5	1.6 (1.42–1.81)	< 0.0001
24–27	0.83 (0.76–0.91)	< 0.0001
Over 27	0.86 (0.78–0.95)	0.002
**Hypertension**		
Normal	1	
Abnormal	0.89 (0.83–0.96)	0.001
**Diabetes**		
Normal	1	
Abnormal	1.02 (0.95–1.1)	0.511
**Cholesterol**		
Normal	1	
Abnormal	0.99 (0.92–1.06)	0.794
**Triglyceride**		
Normal	1	
Abnormal	1.21 (1.11–1.32)	< 0.0001
**Alb**		
Normal	1	
Abnormal	2.59 (2.33–2.89)	< 0.0001
**Gl**		
Normal	1	
Abnormal	0.94 (0.87–1.02)	0.125

**Figure 2:**
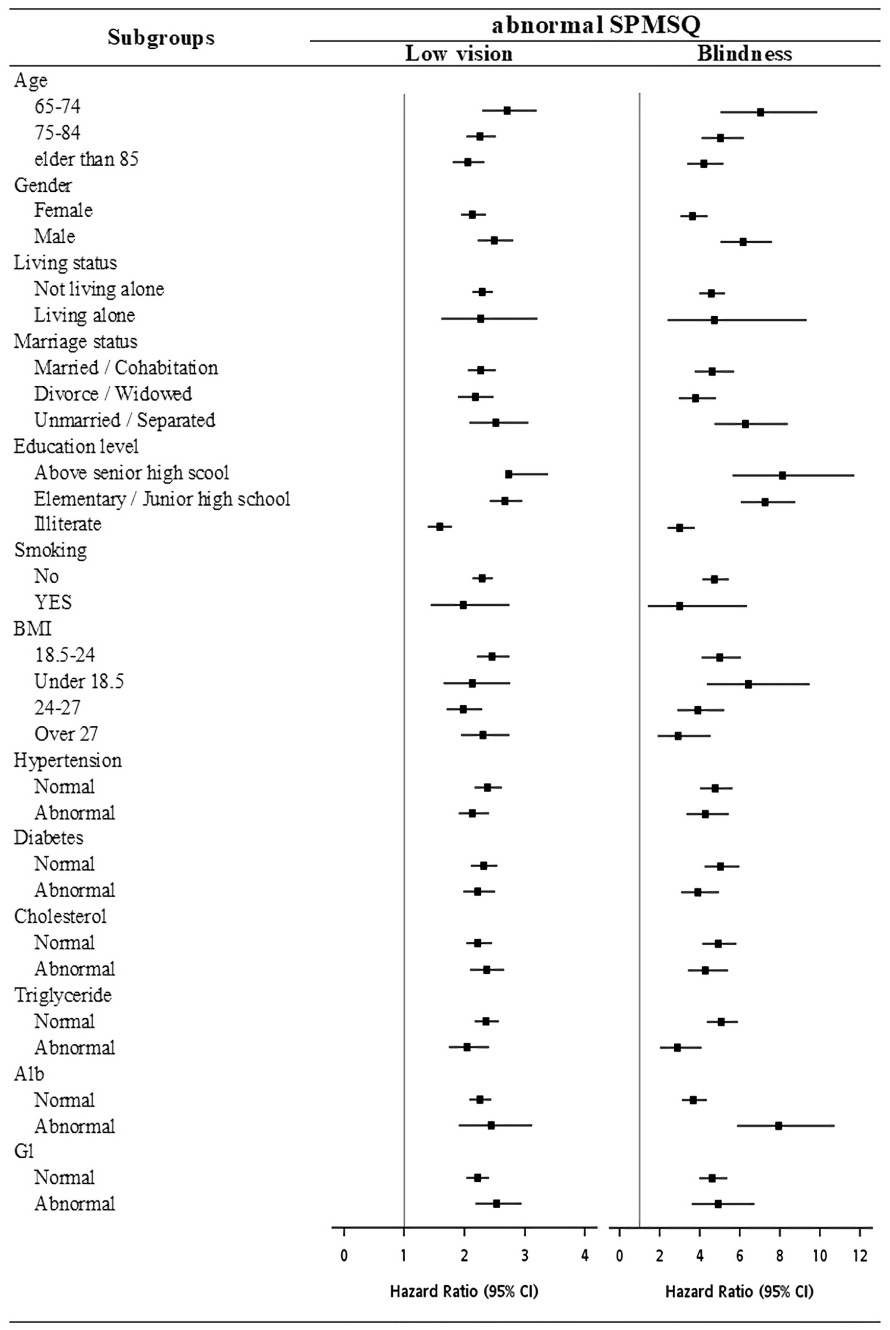
Forest plots of potential cognitive decline, as demonstrated by abnormal SPMSQ scores in each subgroup. Hazard ratios (HRs) were represented by the squares, and the horizontal lines crossing the square stood for the 95% confidence intervals (CIs). The HRs showed similar trends for each subgroup. In each subgroup, those with low vision and blindness were significantly associated with abnormal SPMSQ scores.

The results of the subgroup analyses were shown in Fig. 2. The HRs showed similar trends for each subgroup, that is, the HRs of abnormal SPMSQ for participants with low vision or blindness was significantly higher than those with normal vision. Among the education level above senior high school participants, those with visual impairment showed a significantly high risk of cognitive decline (blindness: HR = 8.13, 95% CI 5.62–11.75; low vision: HR = 2.73, 95% CI 2.73–3.38). Among the underweight (BMI under 18.5) participants, visual impairment participants were significantly associated with increased risk of cognitive decline (blindness: HR = 6.40, 95% CI: 4.33–9.46; low vision: HR = 2.13, 95% CI 1.65–2.75).

## Discussion

In this study, we included 105,208 participants, about one third of the number of elderly people over 65 in Taipei City, over a 7-year period to examine the risk factors related to cognitive decline, as demonstrated by abnormal SPMSQ. We found that visual impairment is associated with cognitive decline, after adjustment for potential confounders. Moreover, we demonstrated that an older age, female, not living alone, single marriage status, low education, no drinking, hypertension, abnormal blood TG and albumin levels were independently correlated with cognitive decline. Our findings help clarify that visual impairment is a risk factor for cognitive decline. Therefore, early prevention and treatment of visual impairment may be an important method to prevent cognitive decline in Asian elderly.

Although many previous studies, mostly conducted in Western populations, have shown that visual dysfunction was related to cognitive decline^10,11^. Zheng et al. suggested that visual acuity has a substantial influence on subsequent change in cognitive function^12^. However, there were still studies showing that there were no correlation between the two. Longitudinal findings of the Blue Mountain Eye Study, including 3654 participants in Australia, showed that by using a modified version of the Mini Mental State Examination (MMSE), no significant association was found between visual impairment and cognitive decline^19^. They believed that the positive correlation between sensory dysfunction and cognitive decline reported in previous studies was due to sensory impairment leading to impaired performance on cognitive function tests. In this study, the SPMSQ was used to screen for possible cognitive decline. It has been validated for use with older Taiwanese adults^20–22^. Kojaie-Bidgoli et al. found that SPMSQ has good validity and reliability in diagnosing cognitive impairment, and can even be used for illiterate patients^23^. Generally, an error score equal to or greater than 3 indicates potential cognitive decline, and the cut-off point can be equal to MMSE score of 23^14^. For the elderly with visual impairment, investigators can verbally ask them during the SPMSQ assessment, which can prevent visual impairment from impairing the performance of the cognitive function test evaluation. Moreover, SPMSQ is easy to use by a healthcare worker and investigators with little training^24^. Therefore, we thought that the impact of visual impairment on the performance of SPMSQ test can be minimized.

Many explanations can explain why visual impairment is related to cognitive decline. Some believe that visual impairment and cognitive decline have common risk factors, such as microvascular pathology and older age, so these factors cause cognitive decline^25–27^. However, in our study, after controlling for possible microvascular risk factors (such as high blood pressure, high blood sugar and cholesterol levels), the correlation between visual impairment and cognitive decline is still obvious, which indicates that visual impairment is one of the factors for cognitive decline. It is possible that elderly people with visual impairment are more likely to have communication difficulties and decline in physical and psychological functions, which may lead to social disconnection or depression, which accelerates brain atrophy, all of which may lead to cognitive decline^9,28,29^.

In addition to visual impairment, we found that an older age, female, not living alone, single marriage status, low education, no drinking, hypertension, abnormal blood TG and albumin levels were independently correlated with cognitive decline. Older age, female, single marriage status, low education, hypertension, malnutrition were well-known factors for cognitive decline^30,31^. However, it is generally believed that living alone causes social isolation and therefore easily leads to cognitive decline. We speculated that the difference was due to the well-developed social welfare in Taiwan. In Taiwan, people with dementia usually do not live alone and are taken care of by nursing homes or relatives. This is why not living alone was significantly associated with cognitive decline^32^. Similarly, people with dementia are less able to drink on their own, which may explain why our study found that not drinking alcohol is associated with cognitive decline.

There were several limitations to this study. The first and main point was that we used general population screening tools SPMSQ instead of comprehensive clinical assessments to verify cognitive decline. In particular, SPMSQ does not have the best ability to detect mild cognitive deficits^33^. Further examination is needed to verify cognitive decline. The second point is that due to communication difficulties, cognitive decline may limit the ability to obtain an accurate assessment of visual acuity. Although we cannot completely alleviate this situation, we excluded participants who cannot complete the vision test due to physical or cognitive impairment. Third, there were many factors related to dementia^30^, but limited by the available data, we could not include all factors in the analysis. Despite these limitations, the strengths of this study included a large population-based cohort design, reasonable follow-up and standardized methods for assessing vision and cognitive decline.

In conclusion, we demonstrated visual impairment was independently and significantly associated with greater incident cognitive decline among elderly Asian persons. Our findings imply that treatment of visual impairment could help to reduce the incidence of cognitive deterioration in the aged population. Further large prospective studies are needed to confirm the possible biological and social mechanisms involved.
